# The Effect of Lifestyle Intervention on Systemic Oxidative Stress in Women with Obesity and Infertility: A Post-Hoc Analysis of a Randomized Controlled Trial

**DOI:** 10.3390/jcm10184243

**Published:** 2021-09-18

**Authors:** Zheng Wang, Arno R. Bourgonje, Henk Groen, Amaal E. Abdulle, Astrid E. P. Cantineau, Anne M. van Oers, Lotte van Dammen, Marian L. C. Bulthuis, Vincent Wekker, Ben W. J. Mol, Tessa J. Roseboom, Harry van Goor, Annemieke Hoek

**Affiliations:** 1 Department of Obstetrics and Gynecology, University of Groningen, University Medical Center Groningen, 9700 RB Groningen, The Netherlands; z.wang@umcg.nl (Z.W.); a.e.p.cantineau@umcg.nl (A.E.P.C.); a.m.van.oers@umcg.nl (A.M.v.O.); L.vanDammen@sanquin.nl (L.v.D.); 2 Department of Gastroenterology and Hepatology, University of Groningen, University Medical Center Groningen, 9700 RB Groningen, The Netherlands; a.r.bourgonje@umcg.nl; 3 Department of Epidemiology, University of Groningen, University Medical Center Groningen, 9700 RB Groningen, The Netherlands; h.groen01@umcg.nl; 4 Department of Internal Medicine, University of Groningen, University Medical Center Groningen, 9700 RB Groningen, The Netherlands; a.eman.abdulle@umcg.nl; 5 Department of Human Development and Family Studies, Iowa State University, Ames, IA 50011, USA; 6 Department of Pathology and Medical Biology, University of Groningen, University Medical Center Groningen, 9700 RB Groningen, The Netherlands; m.bulthuis01@umcg.nl; 7 Department of Obstetrics and Gynecology, University of Amsterdam, Amsterdam UMC, Meibergdreef 9, 1105 AZ Amsterdam, The Netherlands; v.wekker@amsterdamumc.nl (V.W.); t.j.roseboom@amsterdamumc.nl (T.J.R.); 8 Department of Obstetrics and Gynecology, Monash University, 3800 Melbourne, Australia; ben.mol@monash.edu; 9 Department of Clinical Epidemiology-Biostatistics and Bioinformatics, Amsterdam Public Health Research Institute, 1105 AZ Amsterdam, The Netherlands

**Keywords:** lifestyle intervention, obesity, infertility, weight loss, oxidative stress, cardiometabolic health, high-sensitivity C-reactive protein

## Abstract

We aimed to study whether lifestyle intervention could reduce systemic oxidative stress (OS) and the association between OS and cardiometabolic outcomes in women with obesity and infertility. From 2009 to 2012, infertile women with a BMI ≥ 29 kg/m^2^ were randomly assigned to a six-month lifestyle intervention followed by infertility treatment (*N* = 289) or to prompt infertility treatment (*N* = 285). Fasting serum free thiols (FT) concentrations were determined by colorimetry at baseline, at three and six months after randomization. Generalized estimating equations and restricted cubic spline regressions were used to estimate mean differences in serum FT levels between groups and to explore associations between serum FT levels and cardiometabolic outcomes. Baseline serum FT levels did not differ between the two groups (*N* = 203 in the intervention group vs *N* = 226 in the control group, 222.1 ± 48.0 µM vs 229.9 ± 47.8 µM, *p* = 0.09). Body weight decreased by 3.70 kg in the intervention group compared with the control group at six months (95% confidence interval [CI]: −7.61 to 0.21, *p* = 0.06). No differences in serum FT levels were observed between groups at either three months (*N* = 142 vs *N* = 150, mean differences: −1.03 µM, 95% CI: −8.37 to 6.32, *p* = 0.78) or six months (*N* = 104 vs *N* = 96, mean differences: 2.19 µM, 95% CI: −5.90 to 10.28, *p* = 0.60). In a pooled analysis of all available measurements, triglycerides (crude B: 5.29, 95% CI: 1.08 to 9.50, *p* = 0.01), insulin (crude B: 0.62, 95% CI: 0.26 to 0.98, *p* = 0.001), and homeostasis model assessment of insulin resistance (crude B: 2.50, 95% CI: 1.16 to 3.38, *p* < 0.001) were positively associated with serum FT levels. High-sensitivity C-reactive protein (hs-CRP) was negatively associated with serum FT levels (crude B: −0.60, 95% CI: −1.11 to −0.10, *p* = 0.02). The change in hs-CRP during the lifestyle intervention was strongly and inversely associated with serum FT levels (crude B: −0.41, 95% CI: −0.70 to −0.13, *p* = 0.005). No significant deviations from linear associations were observed between serum FT and hs-CRP. We do not observe an improvement in systemic OS in women with obesity and infertility with modest weight loss. There were potential associations between OS and biomarkers of cardiometabolic health. Trial registration: This trial was registered on 16 November 2008 at the Dutch trial register (NTR1530).

## 1. Introduction

Oxidative stress (OS) refers to the imbalance between reactive oxygen species (ROS) production and the ability to scavenge these reactive metabolites [1]. Although ROS are implicated in a myriad of physiological processes [2], pathological overproduction of ROS, as occurs in the case of OS, is considered to be associated with the development of various chronic OS-related diseases, such as obesity, diabetes, chronic obstructive pulmonary diseases, chronic kidney diseases, and cardiovascular diseases (CVD) [3,4]. Recently, the role of OS in the development of CVD has gained significant attention [5,6,7]. In obesity, the level of ROS produced by adipocytes increases due to intracellular fat excess, thereby stimulating the expression and secretion of inflammatory adipokines, which contributes to obesity-associated cardiovascular risk [8]. However, the exact mechanisms leading to an increased obesity-associated cardiovascular risk profiles and the role played by OS in this process remains not fully understood.

OS could be reflected by thiols, which are organosulfur compounds with a free sulfhydryl (R-SH) moiety, occurring both in cells and in extracellular fluids. Free thiols (FT) are thought to protect against OS by scavenging ROS and are active components of the antioxidant buffering capacity in the body, and they are thus regarded as a useful marker for systemic redox status [9]. OS is reflected by reduced levels of serum FT since they are readily oxidized by reactive species [10,11]. For example, previous study confirmed that serum FT levels are lower in patients with Crohn’s disease compared to healthy controls [12]. In addition, higher serum FT levels were indicative of a more favorable disease outcome in patients with heart failure [13], type 2 diabetes mellitus (T2DM) [14], and renal transplant recipients [15]. Kundi et al. [16] were the first to demonstrate that FT can be measured as a relatively cheap and robust OS marker in subjects with acute myocardial infarction, showing that serum FT levels were statistically significantly decreased in patients with acute myocardial infarction as compared to healthy controls.

Lifestyle interventions consisting of dietary caloric restriction, physical activity increment, and behavioral modification comprise the first-line treatment option to reduce the risk of obesity-associated CVD [17,18]. Weight loss through lifestyle interventions improves CVD risk profile: it decreases blood pressure, total cholesterol, triglycerides, and fasting glucose levels, while it increases insulin sensitivity [19,20,21]. The mechanisms underlying the improvement of the cardiometabolic profile after weight loss are not yet fully understood, but reduced OS may play a particular role in this process by increasing the insulin sensitivity of adipocytes [22]. In a lifestyle intervention, not only caloric restriction is essential, but also the quality and composition of the diet strongly influence the cardiometabolic profile [23]. High consumption of vegetables and fruits is related to low OS [24]. Moreover, regular moderate to vigorous exercise seems to ameliorate the whole-body redox status [25]. However, the effects of lifestyle intervention on OS in women with obesity have not yet been thoroughly investigated.

Given the potential of serum FT as a biomarker of OS, the potential effects of lifestyle interventions on systemic OS, and that the interaction between OS and cardiovascular risk profiles have not been completely clarified, we examined (i) whether the lifestyle intervention could reduce OS, (ii) the association between OS and cardiometabolic outcomes, and (iii) whether changes in cardiometabolic markers in response the lifestyle intervention are mirrored by altered OS in women with obesity and infertility.

## 2. Materials and Methods

This study is a post-hoc analysis of the LIFEstyle randomized controlled trial (RCT). The study protocol and main outcomes have been published previously [26,27]. The LIFEstyle trial was registered in the Netherlands Trial Registry (NTR 1530). All procedures were in accordance with the Declaration of Helsinki (2013) and approved by the Institutional Review Board of the University Medical Center Groningen (full name in Dutch: “Medisch Ethische Toetsingscommissie, METc”, no.: 2008/284).

### 2.1. Subjects and Lifestyle Intervention

In the original study, a total of 577 women with obesity and infertility and a BMI ≥ 29 kg/m^2^ (29 was chosen because in a subfertile obese cohort, the chance of becoming pregnant in one year decreases with 4% with every BMI increase above a BMI of 29 [28]) who were between 18–39 years old were included and randomly assigned to a six-month lifestyle intervention followed by infertility treatment or to prompt infertility treatment without lifestyle intervention in the period of June 2009 to June 2012. The main goal of the lifestyle intervention was to achieve a weight loss of at least 5% of their original body weight or a reduction in BMI to at least <29 kg/m^2^ within the intervention period of six months. Once the target was achieved, subjects in the intervention group could receive infertility treatment in case no spontaneous pregnancy occurred during the intervention period. Subjects in the control group received standard care, including infertility treatment (ovulation induction, intra uterine insemination, in vitro fertilization treatment, or intracytoplasmic sperm injection) immediately if needed after randomization.

Lifestyle coaching was developed under the recommendations of the National Institute of Health [29]. Subjects were guided by coaches or dieticians who were trained prior to the study. The intervention consisted of an energy-restricted diet with a reduction of 600 kcal/day on average but with an intake of at least 1200 kcal/day, an increase of physical activity with a goal of 10,000 steps daily monitored by a pedometer, two to three moderate-to-vigorous sessions (60–85% of maximum heart rate frequency) a week, and motivational counselling. The motivational counselling was concentrated on building awareness of lifestyle factors that contribute to the development of obesity.

### 2.2. Clinical and Laboratory Measurements

During the hospital visits at randomization, at three months, and at six months after randomization, body weight (kg), height (cm) and waist and hip circumference (cm) were measured by research nurses who were blinded to the treatment assignment. Fasting blood samples were collected by venipuncture into one serum and one sodium fluoride vacutainer tube. Serum samples were kept at room temperature for coagulation, centrifuged at 4 °C, and then stored at −80 °C. Fasting glucose, insulin, triglycerides, total cholesterol, low-density lipoprotein cholesterol (LDL-C), high-density lipoprotein cholesterol (HDL-C), and high-sensitivity C-reactive protein (hs-CRP) were measured [30]. Homeostasis model assessment of insulin resistance (HOMA-IR) was calculated as fasting insulin concentration (μU/mL) multiplied by fasting glucose concentration (mmol/L) divided by 22.5 Subjects were identified with metabolic syndrome if they met at least three of the following criteria: (1) glucose ≥ 5.6 mmol/L; (2) HDL-C < 1.3 mmol/L; (3) triglycerides ≥ 1.7 mmol/L; (4) waist circumference ≥ 88 cm or (5) blood pressure ≥ 130/85 mmHg [31]. The effects of lifestyle intervention on cardiometabolic health have been published, and the measurement method as well as intra- and inter-assay variation of those outcomes have been elaborately described [30].

Serum concentrations of FT were measured as previously described, but with minor modifications [32,33]. Briefly, serum samples were thawed and four-fold diluted using 0.1 M Tris buffer (pH 8.2). Using the Varioskan microplate reader (Thermo Scientific, Breda, the Netherlands), background absorption was measured at 412 nm, together with a reference measurement at 630 nm. Subsequently, 20 μL of 1.9 mM 5,5′-dithio-bis (2-nitrobenzoic acid) (DTNB, Ellman’s Reagent, CAS number 69-78-3, Sigma-Aldrich Corporation, St. Louis, MO, USA) in 0.1 M phosphate buffer (pH 7.0) were added to the samples. Next, absorbances were measured again after samples were incubated for 20 min at room temperature. Final concentrations of serum FT were established by parallel measurement of an L-cysteine (CAS number 52-90-4, Fluka Biochemika) calibration curve with a concentration range of 15.625–1000 μmol/L in 0.1 M Tris/10 mM EDTA (pH 8.2). Intra- and interday coefficients of variation of all measurements were <10%.

### 2.3. Statistical Analysis

Subjects with at least one serum FT measurement at randomization, three months or six months were included. Data collected from pregnant women was excluded from the analyses.

Baseline characteristics of included subjects and serum FT, anthropometrics, and biochemical measurements at randomization, at three months, or at six months after randomization were expressed as mean ± standard deviation for normally distributed continuous variables, median (interquartile range) for non-normally distributed continuous variables or proportions (percentage, %) for categorical variables. Normality testing was performed using histograms, normal probability plots (Q-Q plots) combined with the Kolmogorov-Smirnov (K-S) test. For continuous variables, the difference between the two groups were assessed with Student’s T-test or Mann–Whitney U-test where relevant, and for categorical variables the Chi-square test was used.

To examine the differences between the intervention group and the control group regarding variations of serum FT levels over time, multilevel analysis (generalized estimating equations) was performed according to the intention-to-treat principle. An exchangeable correlation matrix with a fixed correlation between measurements over time was used. This allowed us to use all available measurements and to correct for within-subject correlations. Baseline serum FT levels, time, group, and the interaction between time and group were included in the model. Since infertility treatment might have potential influence on serum FT levels, an additional analysis was performed adjusted for receiving any type of infertility treatment at the time of blood sample taken.

Generalized estimating equations were also used to examine the overall associations between cardiometabolic outcomes and serum FT levels measured at the various time points. We further investigated the association of serum FT levels with ∆weight/BMI and of serum FT levels with ∆cardiometabolic outcomes which showed significant results in above-mentioned analyses (i.e., triglycerides, hs-CRP, and HOMA-IR) between baseline and three months, during which most of the weight loss occurred. All models were adjusted for baseline serum FT levels. For the calculation of ∆weight/BMI, we first used ∆BMI as continuous measure. Since there was no association between serum FT levels and ∆BMI, we further categorized ∆BMI based on the quartile (≤−1.50; −1.40 ≤ ∆BMI ≤ −0.60; −0.50 ≤ ∆BMI ≤ 0.20; ∆BMI ≥ 0.30) and categorized weight loss based on success (≥5% of original body weight or BMI < 29 kg/m^2^ after randomization). For the calculation of ∆triglycerides, we first used ∆triglycerides as continuous measure and then categorized ∆triglycerides based on the quartile (∆triglycerides ≤ −0.26; −0.25 ≤ ∆triglycerides ≤ −0.01; 0 ≤ ∆triglycerides ≤ 0.25; ∆triglycerides ≥ 0.27). The same methods were applied for the calculation of ∆HOMA-IR (∆HOMA-IR ≤ −0.80; −0.79 ≤ ∆HOMA-IR ≤ −0.08; −0.07 ≤ ∆HOMA-IR ≤ 0.72; ∆HOMA-IR ≥ 0.73). Since there was a statistically significant linear association between FT levels and ∆hs-CRP, we did not categorize ∆hs-CRP into quartiles. Instead, we explored the possibility of a non-linear association between ∆FT and ∆hs-CRP at three months after randomization and between serum FT levels and hs-CRP at each of the timepoints separately using restricted cubic spline regression (five knots). The levels of hs-CRP were log-transformed. In these analyses, we pooled data of all available subjects, regardless of the allocated randomization group, since the effect of randomization group is bidirectional (affecting both cardiometabolic outcomes and OS), and therefore, did not bias the association between cardiometabolic outcomes and serum FT levels.

The Statistical Package for Social Science (IBM SPSS, Armonk, NY, USA, version 25.0) and Stata (Statacorp, College Station, TX, USA, version 14.0SE) were used to perform statistical analyses and GraphPad Prism (San Diego, CA, USA, version 8.0) was used for data visualization. A *p*-value < 0.05 was considered to be statistically significant.

## 3. Results

### 3.1. Study Population Characteristics

**Figure 1:**
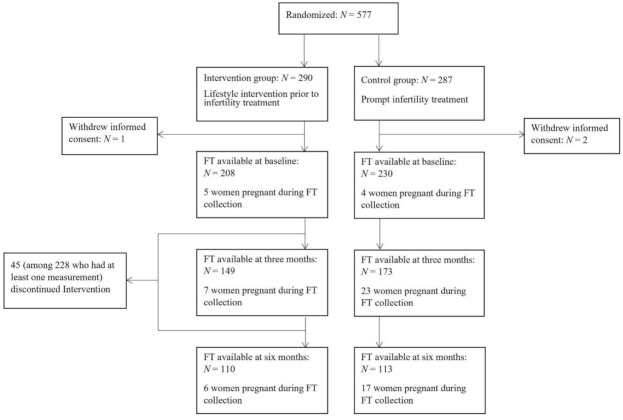
Flow chart of the study. Values are based on the number of subjects for whom serum FT were available. Due to drop-outs, pregnancy, failing to collect blood samples, or running out blood samples in other investigations, the number of available cases decreased over time. Abbreviations: FT: free thiols.

A total of 577 subjects were initially randomized, with 289 subjects were allocated to the intervention group and 285 subjects to the control group (three subjects withdrew informed consent). From the 289 subjects who were allocated to the intervention group, there were 228 (78.9%) subjects who had at least one serum FT measurement (at baseline, at three months or at six months), versus 242 (84.9%) subjects in the control group. A flow chart of the study detailing the number of serum FT measurements is shown in Figure 1. Baseline characteristics of all included subjects are presented in Table 1. The mean age was 30.0 ± 4.5 years with a mean BMI 36.1 ± 3.4 kg/m^2^ in the intervention group, and 29.9 ± 4.6 years and 35.8 ± 3.2 kg/m^2^, respectively, in the control group. There were no statistically significant differences in education and smoking status between the two groups.

Serum FT levels and cardiometabolic outcomes are shown in Table 2. Serum FT values were available for 429 women at baseline (*N* = 203 in the intervention group and *N* = 226 in the control group). Serum FT levels at baseline was 222.1 ± 48.0 µM in the intervention group and 229.9 ± 47.8 µM in the control group without statistically significant difference (*p* = 0.09). Similarly, there were no differences in BMI, cardiometabolic parameters, and the rate of metabolic syndrome at baseline between the two groups. Serum FT levels at three months were 222.4 ± 45.0 µM vs 228.3 ± 43.2 µM in the intervention group (*N* = 142) and the control group (*N* = 150) respectively without statistically significant difference (*p* = 0.26). At three months after randomization, body weight decreased within the intervention group compared to the control group although this difference was not statistically significant (mean difference: −2.75 kg, 95% confidence interval [CI]: −5.84 to 0.34, *p* = 0.08). Concurrently, insulin levels (*p* = 0.006) and HOMA-IR (*p* = 0.005) statistically significantly decreased in the intervention group compared to the control group. There were fewer women with metabolic syndrome in the intervention group than in the control group (46.5% vs 64.8%, OR: 0.47, 95% CI: 0.28 to 0.79, *p* = 0.004). At six months after randomization, serum FT levels were 214.0 ± 40.9 µM in the intervention group (*N* = 104) and 220.4 ± 39.8 µM in the control group (*N* = 96). Body weight was still not statistically significant different between the two groups (mean difference: −3.70 kg, 95% CI: −7.61 to 0.21, *p* = 0.06). There were no statistically significant differences in cardiometabolic parameters (triglycerides, total cholesterol, HDL-C, LDL-C, hs-CRP, and HOMA-IR). The proportion of women having metabolic syndrome statistically significant decreased in the intervention group compared to the control group (41.5% vs 57.8%, OR: 0.52, 95% CI: 0.28 to 0.96, *p* = 0.04).

### 3.2. Effect of the Lifestyle Intervention on OS

**Figure 2:**
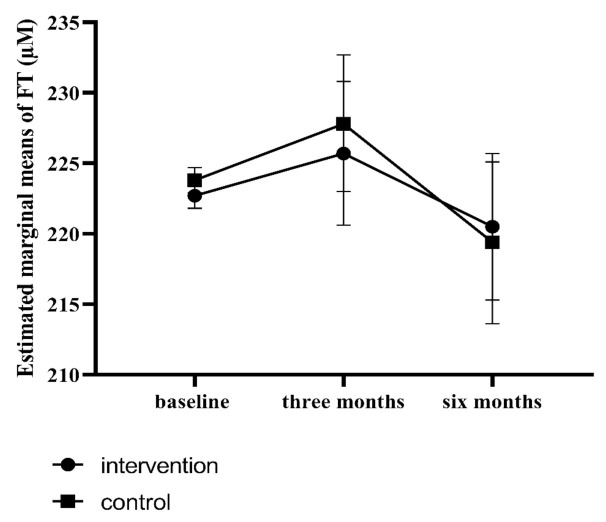
Estimated marginal means from baseline-corrected^ GEE analyses of serum FT levels. Abbreviations: FT: free thiols; GEE: generalized estimating equations. ^ adjusted for baseline serum FT levels.

There were no statistically significant differences in serum FT levels between the intervention group and the control group at either three months (mean differences: −1.03 µM, 95% CI: −8.37 to 6.32, *p* = 0.78) or six months (mean differences: 2.19 µM, 95% CI: −5.90 to 10.28, *p* = 0.60) after randomization. The correction of infertility treatment at either three or six months did not change the results (the effect was not significant) thus we did not add it to the model. Estimated marginal means of serum FT are shown in Figure 2.

### 3.3. Associations between Serum FT Levels and Cardiometabolic Outcomes

In all available measurements regardless of allocated group and time points, women’s age was negatively associated with serum FT levels (crude B: −0.90, 95% CI: −1.73 to −0.07, *p* = 0.04). Triglycerides (crude B: 5.29, 95% CI: 1.08 to 9.50, *p* = 0.01), insulin (crude B: 0.62, 95% CI: 0.26 to 0.98, *p* = 0.001) and HOMA-IR (crude B: 2.50, 95% CI: 1.16 to 3.38, *p* < 0.001) were positively associated with serum FT levels. Conversely, hs-CRP was negatively associated with serum FT levels (crude B: −0.60, 95% CI: −1.11 to −0.10, *p* = 0.02). The results are shown in Table 3.

### 3.4. Associations of Serum FT Levels with ∆weight/BMI, ∆triglycerides, ∆hs-CRP and ∆HOMA-IR (Three Months—Baseline)

The ∆BMI as continuous measure did not significantly affect serum FT levels at three and six months after randomization (crude B: 0.39, 95% CI: −0.82 to 1.59, *p* = 0.53). There were no differences in serum FT levels between the four ∆BMI quartile groups. The largest BMI decrease group did not significantly affect serum FT levels compared to the largest BMI increase group (crude B: −3.15, 95% CI: −8.90 to 2.61, *p* = 0.28) (Table 4). The ∆triglycerides and ∆HOMA-IR as continuous measure or as groups did not significantly affect serum FT levels at three and six months after randomization. The ∆hs-CRP as continuous measure was strongly and inversely associated with serum FT levels (crude B: −0.41, 95% CI: −0.70 to −0.13, *p* = 0.005).

### 3.5. Non-Linear Associations between Serum FT and Hs-CRP

**Figure 3:**
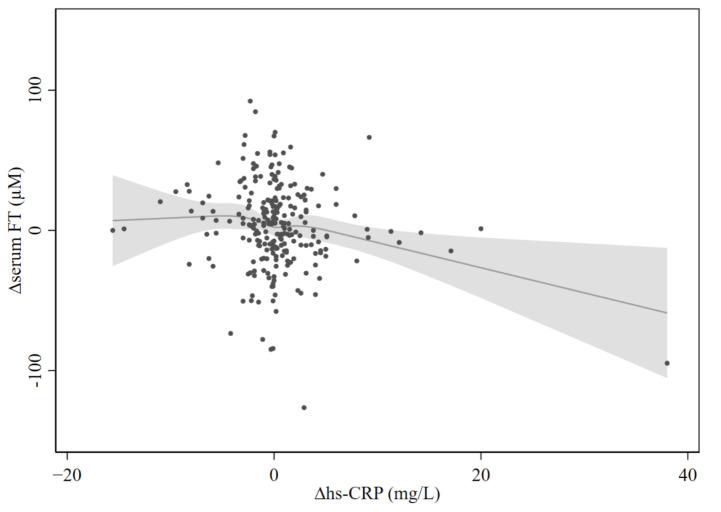
Estimated associations of ∆FT with ∆hs-CRP (three months—baseline) based on restricted cubic splines. The figure shows restricted cubic splines with scatter plot of ∆FT and ∆hs-CRP (three months—baseline). A dot represents one case. The regression line for each spline segment for ∆FT levels is connected with smoothed transitions. The gray area represents the 95% CI. The restricted cubic spline regression does not reveal significant deviations from linear associations. Abbreviations: FT: free thiols; hs-CRP: high-sensitivity C-reactive protein.

**Figure 4:**
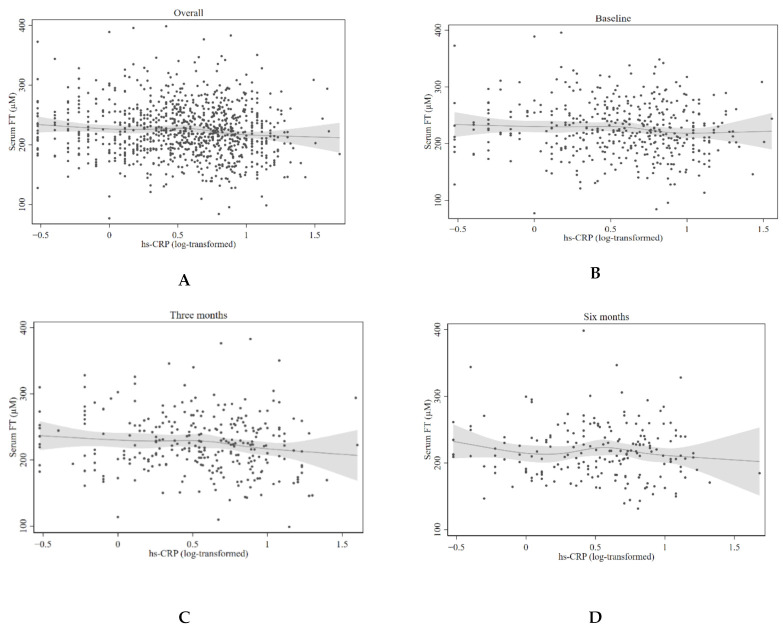
Estimated associations of serum FT with hs-CRP based on restricted cubic splines. The figure shows restricted cubic splines with scatter plot of serum FT and hs-CRP (log-transformed). A dot represents one case. The regression line for each spline segment for serum FT levels is connected with smoothed transitions. The gray area represents the 95% CI. The restricted cubic spline regression does not reveal significant deviations from linear associations. (**A**) Estimated associations of serum FT with hs-CRP (log-transformed) with all available measurements. (**B**) Estimated associations of serum FT with hs-CRP (log-transformed) with baseline measurements. (**C**) Estimated associations of serum FT with hs-CRP (log-transformed) with measurements at three months after randomization. (**D**) Estimated associations of serum FT with hs-CRP (log-transformed) with measurements at six months after randomization. Abbreviations: FT: free thiols; hs-CRP: high-sensitivity C-reactive protein.

No non-linear association of ∆FT with ∆hs-CRP (three months—baseline, *N* = 249, Figure 3) was observed. Besides, restricted cubic spline regression did not reveal any significant deviations from linear associations with serum FT levels and hs-CRP (log-transformed) with measurements at baseline (*N* = 422, *p* = 0.34), three months (*N* = 287, *p* = 0.22), or six months (*N* = 198, *p* = 0.52). The graphs are presented in Figure 4.

## 4. Discussion

In this post-hoc analyses of an RCT including women with obesity and infertility, we aimed to examine whether a six-month lifestyle intervention program could reduce OS represented by serum FT levels, as a derivative for the systemic redox status. However, we did not observe statistically significant differences in serum FT levels between the intervention and the control group during the six months’ follow-up after randomization. The negative association between age and OS was expected, as well as hip circumference (associated with obesity) and OS. Furthermore, we found that triglycerides, insulin, and HOMA-IR were positively associated with serum FT levels and hs-CRP was negatively associated with serum FT levels, while other cardiometabolic outcomes including total cholesterol, HDL-C, LDL-C and glucose were not. Association analyses revealed that ∆hs-CRP during the lifestyle intervention were strongly and inversely associated with variations in serum FT levels but ∆triglycerides or ∆HOMA-IR were not.

A recent observational cohort first demonstrated that bariatric surgery decreases OS in patients with morbid obesity [34]. In that study, 24 patients with a BMI ≥ 40 kg/m^2^ (mean BMI: 44.0 ± 7.03 kg/m^2^) before BS were included. One year after bariatric surgery, the mean BMI was reduced to 28.9 ± 5.1 kg/m^2^, and the degree of OS, as measured by superoxide dismutase, catalase, paraoxonase, and malondialdehyde decreased. In our larger RCT of women with obesity, we did not observe a decrease in OS after three or six months of lifestyle intervention. One possible explanation for the absence of a clear effect of the lifestyle intervention on OS is that the post-hoc analyses might not have sufficient power to detect small differences in serum FT levels at three and six months after randomization. There were 45 (19.7%) women who discontinued the intervention and were incorporated in the analyses according to the intention-to-treat principle, and this might have underestimated the effect of lifestyle intervention on OS. Another possible explanation for the lack of decrease in systemic OS during the intervention could be that subjects were not able to maintain compliance with the intervention, which is a commonly observed phenomenon in the lifestyle intervention programs [35,36]. Consequently, this has led to a rather limited weight loss at six months. Although weight change was limited in the intervention group of the study, the cardiometabolic outcomes were significantly affected at three months after randomization, but not at six months in our previous study [30]. This is in line with the observed OS changes with increased serum FT levels at three months after randomization, whereas at six months serum FT levels decreased. Of note, cardiovascular health in women who successfully lost weight during the intervention improved at the follow-up evaluation at six years after the study [37]. In light of these considerations, future studies are warranted to investigate the effect of lifestyle intervention on systemic OS reflected by relatively cheap and reliable FT measurement. Ideally, performing RCT(s) with long-term lifestyle interventions achieving a more significant decrease in weight loss would enable to further unravel the effect of lifestyle interventions on OS in women with obesity.

Studies concerning the relationship of systemic OS as represented by serum FT levels with cardiometabolic measurements in humans are limited. A study by van Dijk et al. showed that plasma triglycerides were positively and independently associated with FT levels in 168 subjects with or without T2DM [38]. In line with that study, we found a positive association between triglycerides and serum FT levels as well. All subjects included in our study were women with a BMI ≥ 29 kg/m^2^ without severe complications such as hypertension or diabetes, indicating that the relationship observed in our study is not due to T2DM as in the study by van Dijk et al., and that obesity itself might be influencing the relationship between FT and triglycerides. In addition to the association between triglycerides and FT, insulin and HOMA-IR were positively associated with serum FT levels in the current analysis. However, the mechanisms behind these associations remain unclear and warrants further studies to achieve more functional insight.

Moreover, our finding that hs-CRP was negatively associated with serum FT levels aligned with two recent studies in a general population of more than 5000 participants [39,40]. Besides, a study with 223 premenopausal women reported there was a negative association between FT levels and homocistein, a molecule associated with cardiovascular risk such as hsC-RP [41]. In addition, hs-CRP was also significantly and inversely associated with FT in patients with inflammatory bowel disease [12]. hs-CRP is now recognized as a major cardiovascular risk factor and proxy of inflammation [42]. ∆hs-CRP was the only factor among three parameters we examined (∆triglycerides, ∆hs-CRP, and ∆HOMA-IR) that showed association with variations in serum FT levels during the lifestyle intervention in women with obesity. The mechanisms behind the association between OS and inflammation are far from clear. However, the activation of transcription factors and pro-inflammatory genes induced by ROS leads to inflammation. Inflammation causes immune cells to secrete various cytokines and chemokines to recruit other immune cells to OS sites [43]. Consequently, ROS production by immune cells at the site of inflammation increases, leading to OS [44]. All these results underscore that OS and inflammation are associated with obesity-related diseases and that they are not independent of each other.

Strengths of the present study include the well-characterized study population and the prospective, randomized, and longitudinal nature of the data, which enabled us to prospectively evaluate the variations in serum FT levels. However, there are some limitations of the study that need to be taken into consideration. First, the role of OS in female in fertility is far from understood. The available evidence indicates that OS might be a mediator of reproduction disorders, i.e., high OS influences folliculogenesis and corpus lutea function negatively, as well as the oocyte and embryo quality, and thus the fertilization rates, and ultimately lead to infertility [45,46]. The current analysis included women with obesity and infertility seeking infertility treatment. It is not clear how infertility affects our exploration of lifestyle changes on OS and the association between cardiometabolic parameters and OS. Second, the homogeneous study population may also limit generalizability to other populations. Third, the proportion of women of whom there were serum FT measurements available reduced over the course of the study period due to the nature of RCT with drop-outs during the intervention, due to pregnancies that led to exclusion of further physical examination and sampling, due to failure to attend the study visits, or due to blood samples being exhausted in other investigations. To partially overcome this limitation, we compared epidemiological characteristics between subjects who had at least one serum FT measurement at baseline, at three months or at six months after randomization with those who had no serum FT measurements. Results from these analyses indicated that the presented results are representative of the total subjects.

## 5. Conclusions

Our data suggest that a six-month lifestyle intervention does not translate to an improvement in systemic OS estimated by serum FT in women with obesity and infertility with modest weight loss. The association between serum FT and hs-CRP concentrations points to a potential significance of serum FT in the underlying pathophysiological mechanisms of metabolic regulation of inflammation.

## Figures and Tables

**Table 1:** Baseline characteristics of subjects who had at least one serum FT measurement at baseline, at three months or at six months after randomization.

	Total Group (*N* = 470)	Intervention Group (*N* = 228)	Control Group (*N* = 242)	*p*-Value
Age (years)	29.9 ± 4.5	30.0 ± 4.5	29.9 ± 4.6	0.84
Weight (kg)	103.4 ± 13.1	104.2 ± 14.0	102.7 ± 12.1	0.21
BMI (kg/m^2^)	35.9 ± 3.3	36.1 ± 3.4	35.8 ± 3.2	0.23
Western European Ethnicity	411 (87.4%)	200 (87.7%)	211 (87.2%)	0.86
Education				0.54
Primary school	23 (4.9%)	14 (6.1%)	9 (3.7%)	
Secondary education	107 (22.8%)	56 (24.6%)	51 (21.1%)	
Intermediate vocational education	217 (46.2%)	104 (45.6%)	113 (46.7%)	
Advanced vocational education and university	105 (22.3%)	46 (20.1%)	59 (24.4%)	
Unknown	18 (3.8%)	8 (3.5%)	10 (4.1%)	
Current smoker	110 (23.6%)	60 (26.5%)	50 (20.7%)	0.14

Data are presented as mean ± standard deviation or proportions (%).

**Table 2:** Serum FT levels and cardiometabolic outcomes at baseline, at three months, and at six months after randomization.

	Baseline			Three Months			Six Months		
	Intervention Group (*N* = 203)	Control Group (*N* = 226)	*p*-Value	Intervention Group (*N* = 142)	Control Group (*N* = 150)	*p*-Value	Intervention Group (*N* = 104)	Control Group (*N* = 96)	*p*-Value
Serum FT (µM)	222.1 ± 48.0	229.9 ± 47.8	0.09	222.4 ± 45.0	228.3 ± 43.2	0.26	214.0 ± 40.9	220.4 ± 39.8	0.26
Weight (kg)	104.2 ± 14.3	102.6 ± 12.0	0.20	99.8 ± 13.1	102.6 ± 12.5	0.08	98.9 ± 13.0	102.6 ± 13.4	0.06
BMI (kg/m^2^)	36.1 ± 3.4	35.6 ± 3.2	0.13	34.6 ± 3.8	35.5 ± 3.6	0.08	34.7 ± 3.8	35.5 ± 3.6	0.18
Waist circumference (cm)	108.3 ± 9.4	107.1 ± 9.1	0.16	104.3 ± 10.4	105.6 ± 9.4	0.30	104.1 ± 9.9	105.6 ± 10.5	0.30
Hip circumference (cm)	125.4 ± 9.2	124.8 ± 8.8	0.47	121.0 ± 9.5	124.4 ± 9.6	0.004	121.2 ± 9.0	124.8 ± 9.8	0.01
Waist-hip circumference ratio	0.9 ± 0.1	0.9 ± 0.1	0.48	0.9 ± 0.1	0.9 ± 0.1	0.16	0.9 ± 0.1	0.8 ± 0.1	0.31
Triglycerides (mmol/L)	1.2 ± 0.9	1.4 ± 1.0	0.23	1.3 ± 0.8	1.4 ± 1.1	0.34	1.2 ± 1.0	1.5 ± 1.9	0.24
Total cholesterol (mmol/L)	4.8 ± 0.9	4.8 ± 0.8	0.85	4.8 ± 1.0	4.8 ± 0.9	0.80	4.7 ± 0.8	4.9 ± 0.9	0.19
HDL-C (mmol/L)	1.2 ± 0.3	1.2 ± 0.3	0.75	1.2 ± 0.3	1.1 ± 0.3	0.58	1.2 ± 0.3	1.2 ± 0.3	0.47
LDL-C (mmol/L)	3.1 ± 0.8	3.1 ± 0.8	0.73	3.1 ± 0.9	3.1 ± 0.8	0.75	3.1 ± 0.8	3.1 ± 0.8	0.48
hs-CRP (mg/L)	4.2 (2.2; 7.0)	4.0 (1.8; 7.8)	0.61	3.6 (1.7; 7.1)	4.2 (1.9; 7.8)	0.32	3.4 (1.4; 6.5)	3.9 (2.0; 7.4)	0.36
Glucose (mmol/L)	5.3 ± 0.6	5.4 ± 0.7	0.42	5.3 ± 0.6	5.5 ± 0.8	0.09	5.2 ± 0.5	5.4 ± 0.9	0.09
Insulin (pmol/L)	96.1 ± 51.7	101.3 ± 60.6	0.34	90.1 ± 55.7	110.8 ± 70.6	0.006	85.9 ± 52.7	97.4 ± 49.0	0.12
HOMA-IR	3.3 ± 2.0	3.5 ± 2.3	0.34	3.1 ± 2.1	4.0 ± 2.8	0.005	2.9 ± 1.9	3.5 ± 2.1	0.05
Metabolic syndrome	100/193 (51.8%)	124/216 (57.4%)	0.26	54/116 (46.5%)	81/125 (64.8%)	0.004	34/82 (41.5%)	48/83 (57.8%)	0.04

Data are presented as mean ± standard deviation or median (interquartile range) for continuous variables; data are presented as cases/cases available (percentage) for categorical variable. Abbreviations: FT: free thiols; HDL-C: high-density lipoprotein cholesterol; LDL-C: low-density lipoprotein cholesterol; hs-CRP: high-sensitivity C-reactive protein; HOMA-IR: homeostasis model assessment of insulin resistance.

**Table 3:** Associations between serum FT (µM) and cardiometabolic outcomes regardless of randomization group or time points.

	Crude B (95% CI)	*p*-Value
Age (years)	−0.90 (−1.73 to −0.07)	0.04
BMI (kg/m^2^)	−0.71 (−1.61 to 0.20)	0.13
Waist circumference (cm)	−0.14 (−0.42 to 0.13)	0.31
Hip circumference (cm)	−0.50 (−0.82 to −0.18)	0.002
Waist-hip circumference ratio	31.39 (−7.33 to 70.11)	0.11
Triglycerides (mmol/L)	5.29 (1.08 to 9.50)	0.01
Total cholesterol (mmol/L)	1.38 (−2.40 to 5.17)	0.47
HDL-C (mmol/L)	−10.98 (−24.59 to 2.63)	0.11
LDL-C (mmol/L)	−0.73 (−5.35 to 3.89)	0.76
hs-CRP (mg/L)	−0.60 (−1.11 to −0.10)	0.02
Glucose (mmol/L)	3.80 (−0.12 to 7.72)	0.06
Insulin (pmol/L)	0.62 (0.26 to 0.98)	0.001
HOMA-IR	2.50 (1.16 to 3.38)	<0.001
Metabolic syndrome	3.12 (−1.77 to 8.01)	0.21

Abbreviations: FT: free thiols; HDL-C: high-density lipoprotein cholesterol; LDL-C: low-density lipoprotein cholesterol; hs-CRP: high-sensitivity C-reactive protein; HOMA-IR: homeostasis model assessment of insulin resistance; CI: confidence interval.

**Table 4:** Associations between baseline-corrected ^ serum FT (µM) and ∆BMI, ∆triglycerides, ∆hs-CRP and ∆HOMA-IR (three months—baseline).

	Crude B (95% CI)	*p*-Value
**Change in BMI**		
∆BMI as continuous measure	0.39 (−0.82 to 1.59)	0.53
∆BMI categorized		
Method 1: ∆BMI quartiles		
Q1 (∆BMI ≤ −1.50)	−3.15 (−8.90 to 2.61)	0.28
Q2 (−1.40 ≤ ∆BMI ≤ −0.60)	−1.61 (−7.43 to 4.21)	0.59
Q3 (−0.50 ≤ ∆BMI ≤ 0.20)	1.64 (−3.64 to 6.91)	0.54
Q4 (∆BMI ≥ 0.30)	reference	
Method 2: successful weight loss *		
successful	0.22 (−4.40 to 4.84)	0.93
unsuccessful	reference	
**Change in triglycerides**		
∆triglycerides as continuous measure	0.67 (−1.55 to 2.90)	0.55
∆triglycerides categorized (based on quartiles)		
Q1 (∆triglycerides ≤ −0.26)	2.10 (−3.20 to 7.41)	0.44
Q2 (−0.25 ≤ ∆triglycerides ≤ −0.01)	−0.93 (−6.67 to 4.80)	0.75
Q3 (0 ≤ ∆triglycerides ≤ 0.25)	0.13 (−5.47 to 5.74)	0.96
Q4 (∆triglycerides ≥ 0.27)	reference	
**Change in hs-CRP**		
∆hs-CRP as continuous measure	−0.41 (−0.70 to −0.13)	0.005
**Change in HOMA**		
∆HOMA-IR as continuous measure	0.67 (−0.08 to 1.42)	0.08
∆HOMA-IR categorized (based on quartiles)		
Q1 (∆HOMA-IR ≤ −0.80)	−2.63 (−7.26 to 2.01)	0.27
Q2 (−0.79 ≤ ∆HOMA-IR ≤ −0.08)	−4.96 (−10.03 to 0.11)	0.06
Q3 (−0.07 ≤ ∆HOMA-IR ≤ 0.72)	−0.91 (−6.55 to 4.72)	0.75
Q4 (∆HOMA-IR ≥ 0.73)	reference	

Abbreviations: FT: free thiols; hs-CRP: high-sensitivity C-reactive protein; HOMA-IR: homeostasis model assessment of insulin resistance; CI: confidence interval. * ≥ 5% of original body weight or BMI < 29 kg/m^2^ after randomization. ^ All models were adjusted for baseline serum FT levels.

## Data Availability

The data presented in this study are available on request from the corresponding author. The data are not publicly available due to containing sensitive personal information.
